# Farmers’ Intentions to Implement Foot and Mouth Disease Control Measures in Ethiopia

**DOI:** 10.1371/journal.pone.0138363

**Published:** 2015-09-16

**Authors:** Wudu T. Jemberu, M. C. M. Mourits, H. Hogeveen

**Affiliations:** 1 Business Economics Group, Wageningen University, Wageningen, The Netherlands; 2 Department of Veterinary Epidemiology and Public Health, Faculty of Veterinary Medicine, University of Gondar, Gondar, Ethiopia; 3 Department Farm Animal Health, Faculty of Veterinary Medicine, Utrecht University, Utrecht, The Netherlands; The Pirbright Institute, UNITED KINGDOM

## Abstract

The objectives of this study were to explore farmers’ intentions to implement foot and mouth disease (FMD) control in Ethiopia, and to identify perceptions about the disease and its control measures that influence these intentions using the Health Belief Model (HBM) framework. Data were collected using questionnaires from 293 farmers in three different production systems. The influence of perceptions on the intentions to implement control measures were analyzed using binary logistic regression. The effect of socio-demographic and husbandry variables on perceptions that were found to significantly influence the intentions were analyzed using ordinal logistic regression. Almost all farmers (99%) intended to implement FMD vaccination free of charge. The majority of farmers in the pastoral (94%) and market oriented (92%) systems also had the intention to implement vaccination with charge but only 42% of the crop-livestock mixed farmers had the intention to do so. Only 2% of pastoral and 18% of crop-livestock mixed farmers had the intention to implement herd isolation and animal movement restriction continuously. These proportions increased to 11% for pastoral and 50% for crop-livestock mixed farmers when the measure is applied only during an outbreak. The majority of farmers in the market oriented system (>80%) had the intention to implement herd isolation and animal movement restriction measure, both continuously and during an outbreak. Among the HBM perception constructs, perceived barrier was found to be the only significant predictor of the intention to implement vaccination. Perceived susceptibility, perceived benefit and perceived barrier were the significant predictors of the intention for herd isolation and animal movement restriction measure. In turn, the predicting perceived barrier on vaccination control varied significantly with the production system and the age of farmers. The significant HBM perception predictors on herd isolation and animal movement restriction control were significantly influenced only by the type of production system. The results of this study indicate that farmers’ intentions to apply FMD control measures are variable among production systems, an insight which is relevant in the development of future control programs. Promotion programs aimed at increasing farmers’ motivation to participate in FMD control by charged vaccination or animal movement restriction should give attention to the perceived barriers influencing the intentions to apply these measures.

## Introduction

Ethiopia has the largest cattle population in Africa with 54 million heads of cattle [1]. Within the cattle population, foot and mouth disease (FMD) occurs endemically resulting in several outbreaks a year [2]. These outbreaks affect a large part of the country [3] causing significant economic losses in the affected herds [4]. The Ethiopian government is keen in to launching an official control program against FMD to reduce production losses and to improve the export trade of animals and animal products [5].

Successful livestock disease control programs, for example through mass vaccination, depend not only on technical and economic feasibilities, but also on the motivation of the farming community to fully participate in the implementation of the control program [6]. Farmers’ motivation to implement a specific disease control measure is largely driven by their perceptions of the disease’s risk and the effectiveness of available control measures[7,8]. Perceptions about the effectiveness of farming technologies, including that of livestock disease control, are known to be important predictors of the eventual technology uptake [9,10].

Farmers’ adoption of a specific livestock disease control measure or participation in national animal disease control programs involves a behavioral change in the farming practice. In order to achieve behavioral change, insight in the cognitive factors (perceptions) driving the behavior is important. Health behavioral models are often used to study perceptions that influence health related behaviors in public health [11]. Health behavioral models are also being used more and more in animal health research in recent times to study farmers’ behavior with regard to disease control and prevention on their farms [12–16].

Health belief model (HBM) is one of the most commonly used individual health behavioral models in public health [17,18]. The core concept behind the HBM is that health behavior is determined by the personal beliefs or perceptions about the disease’s risk and available control measures [17]. The model has been developed to evaluate perceptions influencing preventive health behaviours (actions undertaken by an individual who believes himself (or herself) to be healthy, for the purpose of preventing or detecting illness in an asymptomatic state) [19]. The basic concept of the HBM also applies to farmers’ behavior related to animal disease prevention in their farms; and has been used to study the factors underlying farmers’ adoption of animal disease prevention and control measures [16].

In Ethiopia, there are three types of cattle production systems, viz. crop-livestock mixed (CLM), pastoral and market oriented systems [20]. Due to differences in the epidemiology and economic impacts of FMD in these production systems [3,4], cattle farmers could have different perceptions about the disease risk and, therefore, different intentions towards the uptake of control measures. Understanding farmers’ perceptions of FMD and its control in the different production systems and their intentions to apply control measures is, therefore, important in designing a national FMD control promotion program that insures the comprehensive participation of all cattle farmers. Currently this understanding is seriously lacking.

The objectives of this study were, therefore, to explore livestock farmers’ intentions to implement FMD control measures in the different cattle production systems of Ethiopia, and to identify perceptions about the disease and its control measures that influence the intentions to implement control measures using the HBM framework.

## Materials and Methods

### 2.1 Theoretical framework

The basic concept of HBM is that health behavior is determined by personal beliefs or perceptions about the disease risk and control measures available to decrease its occurrence [17]. The HBM outlines four basic perception constructs that influence the resulting health behavior. These constructs include:

1. perceived *susceptibility* or belief about the likelihood of getting a disease or condition,
2. perceived *severity* or belief about how serious a condition and its sequelae are,
3. perceived *benefits* or belief in efficacy of the advised action to reduce threat i.e. susceptibility and severity, and
4. perceived *barriers* or belief about the tangible or intangible costs of the advised action.

These perceptions, individually or in combination, explain adoption or non-adoption of a particular health behavior. If individuals believe that they are susceptible to a disease, believe that the disease would have a serious consequences, believe that a prevention measure available to them would be beneficial in reducing their susceptibility to or severity of the disease, or believe the expected benefits outweighs the cost of the available measure, then they are likely to apply the measure. Through time, additional constructs have been added to the model such as self-efficacy (confidence in one’s ability to take action) and cue to action (strategies to activate “readiness”) along with modifying variables (demographic and socio-psychological variables that have an indirect effect on behavior by influencing the perception of susceptibility, severity, benefits, and barriers) [17].

**Fig 1:**
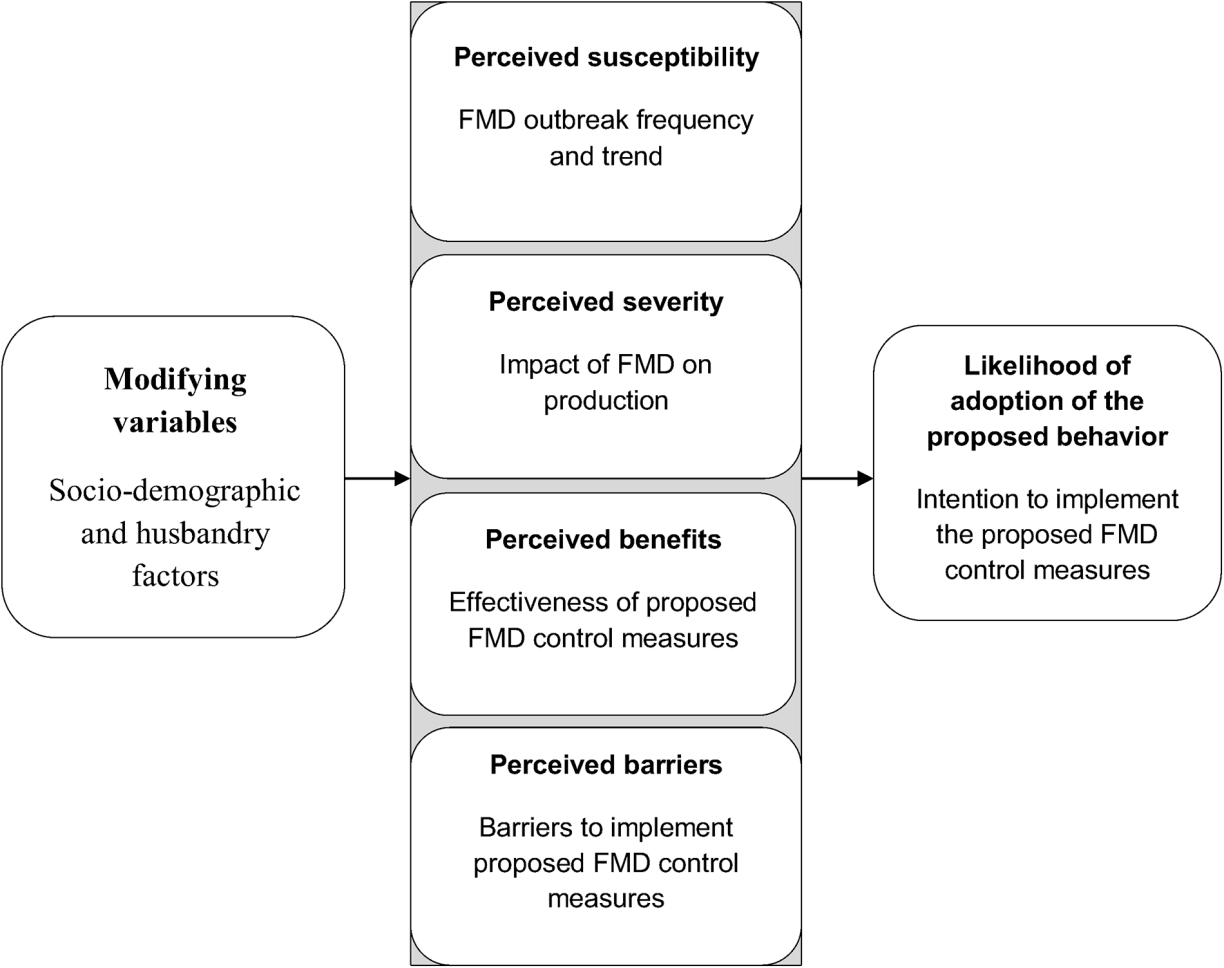
The constructs of the Health Belief Model as applied in the performed analyses on the intention to implement FMD control measures (adapted from Champion and Skinner [17]).

In the present study, the four basic constructs of the HBM were used to assess farmers’ perceptions of FMD and its control in Ethiopia. In the evaluation of the effect of these perceptions on the motivation of farmers to implement control measures against the disease, the *intention* to participate in hypothetical FMD control measures was considered as a proxy of the actual behavior. This is due to absence of any official control in practice to measure the behavior directly. Although intention does not always translate to behavior, it is known to be the immediate and the strongest predictor of behavior [21]. In the analysis, socio-demographics and husbandry variables were used as modifying factors of the perception constructs. The theoretical framework as adapted from Champion and Skinner [17] is presented in Fig 1.

### 2.2 Questionnaire design

A questionnaire was designed based on the framework described above. Each model construct was measured using a set of rating scale items (questions).

The perceived *susceptibility* of farmers to the disease risk was elicited by three rating questions. The first question assessed the frequency of FMD outbreaks as experienced in farmers’ own herd. The second question referred to the experienced frequency in the kebele herd (kebele is the smallest administrative level in Ethiopia). Both questions were answered on a four point rating scale, which indicated the frequency of having an outbreak every 1, 2, 5 or 10 years. In the statistical analysis, this scale was converted into a three points qualitative scale reflecting “high” (corresponding to every year), “medium” (corresponding to every 2 years) and “low” (corresponding to every 5 and 10 years) frequency. The third question measured the perceived trend of FMD occurrence by a three points rating scale (decreasing, unchanging, increasing).

The perceived *severity* was measured using two rating questions on a three points scale (high, medium, and low): one question was about the impact of FMD relative to general livestock production problems and the other question about the impact of FMD relative to the impact of other livestock diseases.

The perceived *benefits* (effectiveness) of potential FMD control measures were measured by two rating scale questions on a three points scale (high, medium, low) for proposed control measures related to vaccination, and herd isolation (avoiding mixing cattle of different household herds) and animal movement restriction.

The perceived *barriers* to the proposed control measures were measured by four rating scale questions on a three points scale (high, medium, low). The four questions referred to the difficulty of paying the costs of vaccination estimated at 40birr/animal/per year for biannual vaccination using trivalent vaccine(1birr = 0.05USD), problem of side effects of vaccination (unwanted effect of the vaccination on animal health), difficulty of trekking and handling animals for vaccination, and the difficulty of herd isolation and animal movement restriction.

The *intentions* were assessed for four proposed specific FMD control measures, including 1) vaccination of cattle two times a year using trivalent vaccine with charge (covering the estimated annual costs of 40 birr per animal) 2) vaccination of cattle two times a year using trivalent vaccine free of charge, 3) herd isolation and animal movement restrictions as a continuous measure (irrespective of the presence or absence of active FMD outbreak) and 4) herd isolation and animal movement restriction only during an outbreak of FMD. Farmers were asked about their intentions to implement these FMD control measures by using yes or no questions.

Socio-demographic variables of farmers such as gender, age and education status, and husbandry variables such as production system, cattle herd size and contribution of livestock to livelihood as well as knowledge of FMD and experience with the disease within own herd were documented using open ended questions.

A pilot survey was tested on 10 farmers and based on the results of this pilot, the questionnaire was adjusted to increase operationalization. The main adjustment consisted of a reduction in the size of the response scale from originally five to three points for the practical reasons of minimizing confusion of farmers and increasing the validity of the rating of the farmers.

### 2.3 Data collection

#### 2.3.1 Cattle production systems

In Ethiopia, the dominant production system is the crop-livestock mixed (CLM) system that prevails in the central highland parts of the country and accounts for about 80–85% of the cattle population and 40% of the land area [20]. In this system, cattle are owned by sedentary farmers who mainly grow crops. Cattle are primarily used for draft power in crop cultivation. The second most dominant farming system is the pastoral production system, which is practiced in the arid and semiarid peripheral parts of the country, and accounts for about 15–20% of the cattle population and 60% of land area [20]. In this system, livestock farming is the main livelihood and cattle are used to produce milk for the family and excess animals for the market. The third type of production system is the market oriented system, which is a small (representing about 1% of cattle population) but growing component of the sector in urban and peri-urban parts of the country. This system primarily consists of dairies with improved breeds and to some extent feedlot operations [20].

#### 2.3.2 Sampling

A sample size of 300 farmers, about 100 farmers from each production system, was chosen for data collection. This sample size was based on a pragmatic consideration of logistic feasibility and reasonable power of test for the intended statistical analyses. The farmers were sampled from four districts (Gondar Zuria, Habru, Gubalafto, Kobo) in the CLM system, from three districts (Dugda Dawa, Yabello and Dire) in the pastoral system and from two cities (Gondar and Addis Ababa) in the market oriented system. Selection of districts and cities was based on the authors’ subjective judgment of representativeness of the production systems and on convenience of accessibility. The difference in the number of districts sampled from the different production systems was a reflection of the proportion of districts in the different production systems.

Because of the absence of any official registration of individual farms and the difference in infrastructure, different approaches were followed in selecting farmers in the different production systems. Sampling of farmers in the CLM and pastoral system was done haphazardly at market places, veterinary clinics and community meetings in the selected districts. In the market oriented system, half of the sub-cities (5 sub-cities) from each city was chosen randomly. The selection of farmers in this system was done in a systematic way by travelling in the streets of the selected sub-cities. Selection of farms started at one end of a randomly selected street and continued by skipping the immediate neighboring farm. This selection procedure was repeated in other streets until the required sample size (10 farmers) for each sub-city was reached.

The questionnaire was prepared in English and translated into Amharic (the local language) for administration (S1 Appendix). It was administered to the selected farmers by face to face interviews. The study proposal was ethically reviewed and approved by the Institutional Review Board of the University Gondar. Oral informed consent was obtained from each participating farmer after reading a written consent form. The use of oral consent was approved by the Institutional Review Board considering the fact that most of the study participants could not read and write to give their consent in writing. The interviewers confirmed the participants’ oral consent by signing on the respective consent form for each interview as per the Board’s guideline. The consent form mainly explains about the purpose of the study, the risks and benefits of participation in the study, conditions of confidentiality, the right to refusal or withdrawal from the study, and has a signature space for confirming the participants informed consent. To avoid response bias, farmers were also told that their responses would not be directly used for action by the government and their responses would be handled anonymously throughout the analysis.

### 2.4 Data analysis

The assessments of the intention of farmers to implement FMD control measures and their perceptions about FMD risk and its control in the different production systems were described using frequencies and percentages.

Analyses of the influence of the perceptions on the intentions to implement the proposed FMD control measures were performed for the two control measures that are considered to be most relevant to the Ethiopian situation: 1) vaccination against FMD two times a year using a trivalent vaccine at the cost of the farmer which is estimated at 40 birr/animal/year (simply referred as vaccination with charge hereafter) and 2) herd isolation and animal movement restriction during an outbreak (simply referred as herd_ iso & mov_res hereafter).

The influence of perceptions on the intentions to implement the two control measures was analyzed separately using multivariable binary logistic regressions. In the analyses, the intentions to implement the control measures (vaccination with charge and herd_ iso & mov_res) were used as dependent variables, and variables that were set to measure the four constructs of HBM as predictor variables. In a formal HBM analysis, the constructs are used as predictor variables in the form of latent variables themselves measured by several items. However, in this study the individual items (or observed variables) (Table 1) were directly used as predictors because of the low internal consistency of the items within the constructs (Cronbach alpha of less than 0.7) [22]. For the intention to implement vaccination with charge, three variables of perceived susceptibility, two variables of perceived severity, one variable of perceived benefit and three variables of perceived barriers were used as predictors. For the intention to implement herd_iso & mov_res, the same set of variables of perceived susceptibility and perceived severity, one variable of perceived benefit, and one variable of perceived barrier were used.

**Table 1:** The perception and modifying factor variables used in the logistic regression analyses and their relation to the HBM constructs.

Variables	Relation to the HBM constructs
Gender	Modifying factors
Age	“
Educational status	“
Production system	“
Cattle herd size	“
Contribution of livestock to livelihood	“
Frequency of FMD occurrence in own herd	Perceived susceptibility
Frequency of FMD occurrence in kebele	“
Trend of FMD outbreak occurrence	“
Impact of FMD relative to other production problems	Perceived severity
Impact of FMD relative to other livestock diseases	“
Effectiveness of vaccination against livestock diseases/FMD	Perceived benefits
Effectiveness of herd_iso & mov_res	“
Cost of FMD vaccination	Perceived barriers
Difficulty of handling animals for vaccination	“
Problem of side effects of vaccination	“
Difficulty of herd_iso & mov_res	“

The effect modifying factors (Table 1) on perceptions about the disease and the studied control measure was analyzed using multivariable ordinal logistic regression due to ordinal scale of the perception variables. In these analyses, perception variables that were found significantly associated with intentions in the multivariable binary logistic regressions were subsequently used as dependent variable and the modifying factors (socio–demographic and husbandry variables) as predictors.

The predictor variables that were measured in three points scale of low, medium and high were treated as categorical variables. Before the regression analyses, presence of collinearity among predictor variables was checked using Spearman correlation coefficient. Correlation coefficients greater than 0.9 were considered to indicate the presence of collinearity [23].The predictor variables were included to the model using the enter method i.e. all the predictor variables are entered at once The fits of the models were assessed using Losmer and Lemeshow test for the binary logistic models and Pearson χ^2^ test for the ordinal logistic models. The Pseudo R square measures (Cox and Snell R square, and Negelkerke R square) were used to assess the predictive power in all models [23]. All statistical analyses were performed using the statistical package SPSS (IBM SPSS Statistics for Windows, Version 22.0. Armonk, NY: IBM Corp).

## Results

The response rate of farmers selected to participate in the study was high; only few of them (<5%) were unable to complete the questionnaire mainly due to time limitations. A total of 293 farmers, 84 from CLM system, 100 from the pastoral system and 109 from the market oriented system completed the questionnaire.

### 3.1 Socio-demographic and husbandry characteristics of the study population

An overview of socio-demographic and husbandry characteristics of the respondent farmers is provided in S1 Table. The majority (85%) of the respondents were male. Relatively, there were more female respondents in the market oriented system than in the other systems. The age of respondents was roughly normally distributed. For most (98%) farmers in the pastoral system livestock keeping was the main livelihood, whereas in the other systems the contribution of livestock to their livelihood was partial or minor. Cattle were the most important species of livestock for all farmers in all systems with the exception of two farmers in the pastoral system, where other species (camel and small ruminants) took prominence.

### 3.2 Farmers’ intentions to implement FMD control, and their perceptions about the disease and its control measures

#### 3.2.1 Intentions to implement FMD control measures

Farmers’ intentions for the proposed control measures are documented in Table 2. If an official vaccination program would be launched against FMD, the majority of pastoral (94%) and market oriented (92%) farmers had a positive intention to vaccinate their cattle at their own costs. However, this intention was much lower (42%) among the farmers in the CLM system. If the vaccination would be given for free, almost all farmers (99%) had the intention to vaccinate their animals. If herds would have to be continuously isolated and animal movement restricted, only 2% of pastoral and 18% of CLM farmers had the intention to comply with this measure. If this measure would be taken only during an outbreak of FMD, 50% of the CLM farmers and 11% of the pastoral farmers had the intention to comply with the measure. The majority of market oriented farmers (>80%) indicated a positive intention to comply with herd isolation and animal movement restriction measures, both in a continuous way and during an outbreak.

**Table 2:** Farmers’ intensions to implement FMD control measures in the different production systems.

FMD control measures	Response	CLM		Pastoral		Market oriented	
		^a^N	%	N	%	N	%
Vaccination with charge	yes	30	42	94	94	70	92
	no	42	58	6	6	6	8
Vaccination free of charge	yes	70	97	100	100	76	100
	no	2	3	0	0	0	0
Herd isolation and animal movement restriction continuously	yes	13	18	2	2	63	83
	no	59	82	98	98	13	17
Herd isolation and animal movement restriction during an outbreak	yes	36	50	11	11	65	86
	no	36	50	89	89	11	14

^a^N = number of farmers

#### 3.2.2 Perceived susceptibility to and severity of FMD

Farmers’ perceived susceptibility to and severity of FMD within the various production systems are documented in Table 3. All pastoralists, 86% of the CLM farmers and 74% of the market oriented farmers did know the disease FMD, and 97%, 93% and 63% of the farmers who knew the disease in the respective systems, experienced the disease in their own herd. Most farmers (78%) in the pastoral system reported an annual occurrence of FMD in their herds. Nonetheless, the majority of the CLM farmers (67%) and market oriented farmers (55%) reported a frequency of every five and two years, respectively. The frequency of occurrence of FMD at the level of their kebeles followed a similar trend. The majority (91%) of farmers in the pastoral system perceived that the frequency of FMD occurrence had increased through time. However, the majority of farmers in the CLM (66%) and market oriented (66%) systems perceived that the frequency of occurrence had not changed through time.

**Table 3:** Farmers’ perceived susceptibility to and severity of FMD in the different production systems.

Variables	Response	CLM		Pastoral		Market oriented	
		N	%	N	%	N	%
**Perceived susceptibility**							
Frequency in own herd, one occurrence in	1 year	3	5	76	78	7	15
	2 years	12	20	16	17	26	55
	5 years	40	67	5	5	7	15
	10 years	5	8	0	0	7	15
Frequency in kebele, one occurrence in	1 year	6	10	90	90	14	19
	2 years	11	17	10	10	44	59
	5 years	42	67	0	0	11	14
	10 years	4	6	0	0	6	8
Trend of occurrence	increasing	4	6	91	91	10	13
	unchanging	43	66	9	9	50	66
	decreasing	18	28	0	0	16	21
**Perceived severity**							
Impact relative to other production problems	low	62	86	12	12	33	43
	medium	9	13	45	45	34	45
	high	1	1	43	43	9	12
Impact relative to other disease problems	low	30	42	13	13	8	10
	medium	30	42	43	44	50	66
	high	12	16	42	43	18	24

The impact of FMD relative to production problems in livestock farming was perceived as low by majority of CLM farmers (86%) and as medium by the majority of the pastoralists (45%) and market oriented farmers (45%). When the FMD impact was compared with the consequences of other livestock diseases, it was perceived as medium by the majority of farmers. However, the overall tendency in the CLM was towards a low perceived impact while in the pastoral and market oriented systems it was towards a high perceived impact.

#### 3.2.3 Perceived benefits and barriers of FMD control measures

The farmers’ perceived benefits and barriers of potential FMD control measures such as vaccination, and herd_iso & mov_res are documented in Table 4. Most farmers in all production systems perceived vaccination as a highly effective disease control measure. The majority of farmers in all three systems considered herd_iso & mov_res also as highly effective in controlling FMD but absolute proportions were smaller than those of vaccination (Table 4).

**Table 4:** The farmers’ perceived benefits and perceived barriers of implementation of potential FMD control measures in the different production systems.

Variables	Response	CLM		Pastoral		Market oriented	
		N	%	N	%	N	%
**Perceived benefits**							
Effectiveness of vaccination	low	0	0	1	1	0	0
	medium	2	3	1	1	21	28
	high	70	97	98	98	55	72
Effectiveness of herd_iso & mov_res	low	9	12	3	3	1	1
	medium	10	14	24	24	18	24
	high	53	74	73	73	57	75
**Perceived barriers**							
Cost of vaccination	low	10	14	30	30	22	29
	medium	3	4	26	26	25	33
	high	59	82	44	44	29	38
Difficulty of trekking and handling animals for vaccination	low	69	96	33	33	29	38
	medium	3	4	32	32	21	28
	high	0	0	35	35	26	34
Problem of side effects of vaccination	low	69	96	100	100	52	69
	medium	2	3	0	0	23	30
	high	1	1	0	0	1	1
Difficulty of herd_iso & mov_res	low	19	27	8	8	26	34
	medium	6	8	19	19	37	49
	high	47	65	73	73	13	17

The majority of farmers; 82% in the CLM system, 44% in the pastoral system, and 38% in market oriented system perceived the costs of vaccination as high. Nonetheless, still a significant proportion of pastoral farmers (26%) and market oriented farmers (33%) considered the costs of FMD vaccination as medium. The difficulty of trekking animals to vaccination centers (when needed) and handling animals for vaccination was perceived as low by the majority (96%) of CLM farmers and market oriented farmers (38%), but high by the majority of pastoral farmers (35%). Most farmers perceived problem of side effects vaccination as low. Difficulty of herd_iso & mov_res was perceived as high by the majority of farmers in pastoral system (73%) and CLM system (65%). Most market oriented farmers considered the difficulty of applying herd_iso & mov_res as medium or low.

### 3.3 Perceptions affecting the intentions to implement FMD control measures

Farmers’ perceived cost of vaccination (perceived barrier) was identified as the most important perception that was significantly associated with the intention to implement vaccination with charge (Table 5). The odds of the intention to implement vaccination with charge was significantly lower in respondents who perceived the cost of vaccination as high (OR (95%CI) = 0.04 (0.004–0.32), P = 0.004) than of those who perceived the cost of vaccination as low.

**Table 5:** Perception variables significantly associated with farmers’ intention to apply vaccination with charge.

Perception variable	Levels	Coefficients	Standard Error	Odds Ratio (95%CI)	P-value
Vaccination cost					0.002
	high	-3.30	1.10	0.04 (0.004–0.32	0.004
	medium	-0.37	1.54	0.69 (0.034–14.26)	0.739
	low	1*	1	1	

* The value 1 in the row represents the reference category of the categorical variable.

Number of data points = 233; Model fit: Hosmer and Lemeshow test χ^2^ = 4.88, df = 8, P = 0.777; Psuedo R square: Cox-Snell R square = 0.351; Nagelkerke R square = 0.358).

Perceived difficulty (perceived barrier) was again found to be the most important perception that was significantly associated with the implementation of herd_iso & mov_res measure (Table 6). The odds of intention to implement herd_iso & mov_res was significantly lower for farmers with high perceived difficulty (OR (95%CI) = 0.05 (0.02–0.14), P < 0.001) than for those with a low perceived difficulty of implementing the measure. Perceived frequency of outbreaks in the kebele herds (perceived susceptibility) and perceived effectiveness of herd_iso & mov_res measure (perceived benefit) were also significantly associated with intention to apply herd_iso & mov_res measure (Table 6). The odds of intention to implement herd_iso & mov_res was significantly higher for farmers with medium perceived susceptibility (OR (95% CI) = 3.92 (1.20–12.70), P = 0.023) than farmers with low perceived susceptibility to FMD. Similarly the odds of intention to implement this measure was significantly higher for farmers with high perceived effectiveness (OR (95% CI) = 3.36(1.12–10.02), P = 0.030) and medium perceived effectiveness (OR (95% CI) = 3.47(1.11–10.87), P = 0.033) than farmers with low perceived effectiveness of herd_iso & mov_res measure.

**Table 6:** Perception variables significantly associated with farmers’ intention to apply herd_iso & mov_res control measures.

Perception variable	Levels	Coefficients	Standard Error	Odds Ratio (95%CI)	P-value
Frequency of outbreaks in kebele					0.009
	high	- 0.23	0.62	0.79 (0.23–2.70)	0.712
	medium	1.37	0.60	3.92 (1.20–12.70)	0.023
	low	1*	1	1	
Herd_iso &mov_res effectiveness					0.048
	high	1.21	0.56	3.36 (1.12–10.02)	0.030
	medium	1.25	0.58	3.47 (1.11–10.87)	0.033
	low	1	1	1	
Herd_iso & mov_res difficulty					<0.001
	high	-3.02	0.52	0.05(0.02–0.14)	<0.001
	medium	-0.70	0.59	0.50(0.16–1.59)	0.238
	low	1	1	1	

* The value 1 in the rows represents the reference category of the categorical variables.

Number of data points = 233; Model fit: Hosmer and Lemeshow test χ^2^ = 2.96, df = 8, P = 0.937; Psuedo R square: Cox-Snell R squar e = 0.482; Nagelkerke R square = 0.645)

### 3.4 Modifying factors that influence perceptions that are significantly associated with intentions to implement FMD control measures

Production system and age of the farmers were the modifying factors that significantly affected the perception of cost of vaccination which was the only significant perception that influenced intention to implement vaccination with charge measure (Table 7). There was a significantly lower odds for farmers in the market oriented system (OR (95%CI) = 0.13 (0.05–0.35), P<0.001) and pastoral system (OR (95%CI) = 0.10 (0.03–0.34), P<0.001) to perceive the costs of FMD vaccination as high than for farmers in the CLM system. An increase in the age of the farmers was observed to significantly increase the odds of perceiving the cost of FMD vaccination as high (OR (95% CI) = 1.03(1.01–1.05), P = 0.015) i.e. an increase in the age of farmers by one year increases the odds of perceiving the cost of vaccination as high by 3%.

**Table 7:** Modifying factors that affect perceptions significantly associated with intentions to implement FMD control measures*.

Factors	Levels	Coefficients	Standard Error	Odds Ratio (95% CI)	P- value
**Cost of FMD vaccination** ^**a**^					
					<0.001
Production system	Market oriented	-2.03	0.50	0.13 (0.05–0.35)	<0.001
	Pastoral	-2.34	0.65	0.10 (0.03–0.34)	<0.001
	CLM	1**	1	1	
Age (years)		0.03	0.01	1.03(1.00–1.05)	0.015
**Frequency of outbreak occurrence in kebele** ^**b**^					
Production system					<0.001
	Market oriented	1.80	0.54	6.06 (2.09–17.60)	0.001
	Pastoral	5.50	0.83	244.21 (48.48–1230.34)	<0.001
	CLM	1	1	1	
**Herd_iso & mov_res effectiveness** ^**c**^					
Production system					<0.001
	Market oriented	0.26	0.56	1.30 (0.44–3.85)	0.641
	Pastoral	-2.70	0.67	0.07 (0.02–0.25)	< 0.001
	CLM	1	1	1	
**Herd_iso & mov_res difficulty** ^**d**^					
Production system					<0.001
	Market oriented	-1.75	0.47	0.17 (0.07–0.45)	<0.001
	Pastoral	0.57	0.61	1.77 (0.54–5.79)	0.349
	CLM	1	1	1	

*The table contains four models for the four significant perception variables as presented in Tables 5 and 6.

Reference marks a, b, c and d provide additional information for each of the four models.

** The value 1 in the rows represents the reference category of the categorical variables.

^a^ Number of data points (N) = 237; Model fit: Pearson χ^2^ = 361.1 df = 360, P = 0.474; Psuedo R square: Cox-Snell R square = 0.172; Nagelkerke R square = 0.198).

^b^ N = 227; Model fit: Pearson χ^2^ = 341.6, df = 346,P = 0.557; Psuedo R square: Cox-Snell R square = 0.537; Nagelkerke R square = 0.611).

^c^ N = 237; Model fit: Pearson χ^2^ = 400.5, df = 360,P = 0.069; Psuedo R square: Cox-Snell R square = 0.529; Nagelkerke R square = 0.603).

^d^ N = 237; Model fit: Pearson χ^2^ = 355.5, df = 360,P = 0.557; Psuedo R square: Cox-Snell R square = 0.212; Nagelkerke R square = 0.244).

Production system was the only modifying factor that significantly affected the perceptions that were significantly associated with intention to apply herd_iso & mov_res measure (perceptions of susceptibility, benefits and barriers) (Table 7). The odds of perceiving higher susceptibility (in terms of frequency of outbreak occurrence in kebele) was significantly higher in the market oriented system (OR (95%CI) = 6.06 (2.09–17.60), P = 0.001) and in the pastoral system (OR (95%CI) = 244.21 (48.48–1230.34), P < 0.001) than in the CLM system. The odds of perceiving higher benefit (perceived effectiveness of herd_iso & mov_res measure) was significantly lower in the pastoral system (OR (95%CI) = 0.07 (0.02–0.25), P <0.001) than in the CLM system. The odds of perceiving higher barrier (perceived difficulty of herd_iso & mov_res measure) was significantly lower in the market oriented system (OR (95%CI) = 0.17 (0.07–0.45), P < 0.001) than in the CLM system.

## Discussion

### 4.1 Intentions to implement FMD control measures

In this study, farmers’ intentions to implement different proposed FMD control measures were explored. Almost all farmers had the intention to vaccinate their cattle against FMD if vaccination would be given free of charge. If the vaccination would be given with charge, the intention to vaccinate decreased to some extent in the pastoral and market oriented system and significantly in the CLM system. This indicates that if full participation of farmers is to be achieved in a vaccination campaign against FMD, the problem with the vaccination cost has to be addressed, particularly for the CLM system where vaccination cost appeared very constraining. This could be done through subsidies or rigorous extension to convince CLM farmers about the cost effectiveness of vaccination. However, the benefit of vaccination at herd level for the CLM could be marginal or nonexistent as inferred from cost per outbreak estimations and disease outbreak incidence studies by Jemberu et al. [3,4]

Farmers’ intention to implement herd_iso & mov_res both as a continuous measure and as measure only during outbreaks varies among the production systems. The intention was low for subsistence systems (CLM and pastoral) but high for market oriented system. Given the current husbandry practice in the subsistence systems, which is dependent on common grazing and watering areas, a continuous implementation of herd_iso & mov_res would be unrealistic especially in the pastoral system. However, implementing this measure at least during outbreaks is of vital importance to control highly contagious disease like FMD and needs strong extension work.

### 4.2 Perceptions significantly influencing the intentions to implement FMD control measures

In the performed analyses, the most important factor identified to significantly affect the intention to implement vaccination against FMD with charge was the perceived barrier (cost of vaccination). This is consistent with literature which indicates that among the four HBM variables (constructs), the perceived barrier has been the most powerful in predicting behavior across various study designs and behaviors [17,19,24,25]. Previous observations in animal disease control programs also showed that uptake of livestock vaccination is cost sensitive, especially in farmers of developing countries [26]. Subsidizing the cost of FMD vaccine may be needed to ensure wide participation of farmers in vaccination programs to achieve sufficient herd immunity for controlling the disease. The conventional FMD vaccine in use is one of the most expensive livestock vaccines [27], and reducing its cost should be an area of future research to increase the success in global control of the disease.

Like in vaccination, the intention to implement herd_iso & mov_res was significantly influenced by perceived barrier (difficulty of herd_iso & mov_res) indicating the importance of perceived barrier in predicting health related behavior. The difficulty of herd isolation and movement restriction is inherent to the husbandry system of traditional extensive livestock production. This problem could only be improved by modernizing the husbandry system and by gradually ending communal grazing practice. Perception of susceptibility (high frequency of FMD outbreak occurrence) was also found associated with positive intention to implement herd_iso & mov_res measure and this was in agreement with common findings that perception of susceptibility is an important predictor of preventive health behavior [19]. Providing information about the risk of the disease to the farmers will encourage the desired behavior with regard to FMD control.

The perceived barrier (cost of vaccination) for vaccination with charge control measure was in turn significantly influenced by the type of production system and age of the farmers. Vaccination cost was perceived high by CLM farmers (high perceived barrier) as compared to the farmers in the other systems which could be due to the relatively lesser importance of livestock in their livelihood. Any FMD control program using vaccination in Ethiopia needs to address the vaccine cost problem in this system which is the dominant production system in the country. Increase in age was associated with increased perception of high cost of vaccination. Literature on agricultural technology adoption mostly indicated younger farmers to be more open to adopt farming technologies than older farmers [10,28].

The production system was also the most important factor that modified the perceptions about the herd_iso & mov_res measure. Perception of difficulty of herd_iso & mov_res was higher for subsistence systems compared to the market oriented system. This is obviously related to the farming system in which they mostly use communal grazing area and watering points, and in case of pastoral systems, have to move from place to place in search of pasture. Given the specific characteristics of the subsistence systems the use of movement control as disease control measure will continue to be a challenge in Ethiopia.

## Conclusions

In this study farmers’ intentions to implement potential FMD control measures and their perceptions about the disease and its control measure are explored, the perceptions that have significant influence on the control intentions are identified, and the socio-demographic and husbandry factors that modify these perceptions are recognized. Farmers’ intentions to implement the proposed FMD control measures except for free vaccination are generally low with some variation among the production systems. The perceptions of barriers such as cost of vaccination and difficulty of herd isolation and movement restriction were found as the most important perceptions that significantly influenced the intentions to implement FMD control measures. These perceived barriers should be targeted to increase farmers’ participation in official disease control programs. The type of production system was seen as the main factor that influenced the relevant perceptions and hence indirectly the intentions to implement the control measures. Promotion programs aiming at increasing farmers ‘motivation to participate in FMD control should, therefore, benefit from the insights in the differences in perceptions among the production systems.

## Supporting Information

S1 Table Socio-demographic and husbandry characteristics of sampled farmers in the different production systems. (DOCX)

S1 Appendix Questionnaire on farmers’ perceptions and intentions to implement foot and mouth disease control measures. (DOCX)
