# Supplementary material for: Farmers’ Intentions to Implement Foot and Mouth Disease Control Measures in Ethiopia
Source: PLoS One. 2015 Sep 16;10(9):e0138363. doi: 10.1371/journal.pone.0138363 (PMC4572705; doi:10.1371/journal.pone.0138363)
Supplement: S1 Table — (DOCX) [file pone.0138363.s001.docx]

**S1 Table. Socio-demographic and husbandry characteristics of sampled farmers in the different production systems.**

| **Variables** | **Levels** | **CLM** | | **Pastoral** | | **Market oriented** | |
| --- | --- | --- | --- | --- | --- | --- | --- |
|  |  | N^a^ | % | N | % | N | % |
| **Age (years)** | 20-30 | 7 | 8 | 43 | 43 | 4 | 4 |
|  | 31-40 | 19 | 23 | 23 | 23 | 34 | 31 |
|  | 41-50 | 25 | 30 | 15 | 15 | 38 | 35 |
|  | 50-60 | 11 | 13 | 11 | 11 | 13 | 12 |
|  | >60 | 22 | 26 | 8 | 8.0 | 19 | 17 |
| **Gender** | F | 1 | 1 | 13 | 13 | 28 | 26 |
|  | M | 83 | 99 | 87 | 87 | 81 | 74 |
| **Education** | none | 36 | 43 | 78 | 78 | 8 | 7 |
|  | primary | 44 | 53 | 19 | 19 | 48 | 45 |
|  | secondary | 3 | 4 | 3 | 3 | 43 | 40 |
|  | tertiary | 0 | 0 | 0 | 0 | 8 | 8 |
| **Cattle herd size** | 1-10 | 72 | 92 | 12 | 12 | 79 | 75 |
|  | 11-20 | 4 | 5 | 35 | 35 | 26 | 25 |
|  | 21-30 | 0 | 0 | 21 | 21 | 0 | 0 |
|  | 30-40 | 2 | 3 | 8 | 8 | 0 | 0 |
|  | >40 | 0 | 0 | 14 | 14 | 0 | 0 |
| **Livestock as source of livelihood** | main | 10 | 12 | 98 | 98 | 30 | 27 |
|  | partial | 7 | 8 | 2 | 2 | 51 | 47 |
|  | minor | 67 | 80 | 0 | 0 | 28 | 26 |
| **Cattle as main species** | yes | 84 | 100 | 98 | 98 | 109 | 100 |
|  | no | 0 | 0 | 2 | 2.0 | 0 | 0 |

^a^N= number of farmers
