# Supplementary material for: Farmers’ Intentions to Implement Foot and Mouth Disease Control Measures in Ethiopia
Source: PLoS One. 2015 Sep 16;10(9):e0138363. doi: 10.1371/journal.pone.0138363 (PMC4572705; doi:10.1371/journal.pone.0138363)
Supplement: S1 Appendix — (DOCX) [file pone.0138363.s002.docx]

**S1 Appendix. Questionnaire on farmers’ perceptions and intentions to implement foot and mouth disease control measures.**

1. ***Farm profile***
   1. Production system: Crop-livestock mixed / Pastoral / Market oriented
   2. Name of the respondent (optional) ____________________
   3. Sex: male / female
   4. Age (years) ____________________
   5. Educational status (illiterate - higher education) ____________________
   6. Experience in livestock keeping (years) ____________________
   7. Livestock owned (species and number) ____________________
   8. Number of cattle owned by age group and purpose:

Cow __________ ____________________

Ox/bull __________ ____________________

Young bull/ heifers __________ ____________________

Calves __________ ____________________

- 1. Contribution of livestock to livelihoods

Major / Partial / Minor

- 1. The livestock species that has a main contribution to the livelihoods (give order)

Cattle / Small ruminants / Camel / Equines / Poultry

1. ***Foot and mouth disease risks / susceptibility***
   1. Have your ever heard of a livestock disease called FMD?

Yes No

- 1. If yes, can you describe it (symptoms and epidemiological features)?

*Continue with questionnaire if farmer knows FMD by describing one or more of the following features*

- - 1. *Lameness ( foot lesion) and salivation (mouth lesion) in cattle and/ or small ruminants*
    2. *Foot lesion in cattle and/ or small ruminants that is contagious*
    3. *Mouth lesion in cattle and/ or small ruminants that is contagious*
    4. *Lameness or mouth lesion, and blisters (sores)on teats of cows*

- 1. Have you ever had an FMD outbreak in your cattle herd ?

Yes / No

- 1. If so, when was the last outbreak you experienced?

this year / last year / 2 years ago / 3 years ago / before 5 years

- 1. How frequently did an FMD outbreak occur in your herd during the last ten years?

every year / every two year / every five year / every ten year

- 1. Have you ever had an FMD outbreak in your kebele?

Yes / No

- 1. When was the last outbreak you experienced in the kebele?

this year / last year / 2 years ago / 3 years ago / before 5 years

- 1. How frequently did an FMD outbreak occur in your kebele during the last ten years?

every year / every two year / every five year / every ten year

- 1. The trend in the occurrence of FMD is in the last five years

decreasing / unchanging / increasing

1. ***Impacts of FMD***
   1. The problem of foot and mouth disease in cattle production as compared to all other (disease and non- disease) production problems is considered

low / medium / high

- 1. The problem of foot and mouth disease in cattle production as compared to all other disease problems in cattle is

low / medium / high

**IV. *Effectiveness of FMD control measures***

- 1. Effectiveness of vaccination to prevent FMD (or any other livestock disease)?

low / medium / high

- 1. Effectiveness of restricting movement and avoiding mixing of your herd with other herd in preventing an introduction of FMD?

low / medium / high

1. ***Possible barriers to implement FMD control***
   1. Difficulty to vaccinate against FMD at a cost of 40 birr/year/per animals

low / medium / high

- 1. Difficulty to trek and handle every animal of your herd two times a year for vaccination

low / medium / high

- 1. Side effects of vaccination against FMD (any cattle disease)

low / medium / high

- 1. Difficulty to restrict movement and to avoid mixing of animals during a FMD outbreak (for a period of 3 months)

low / medium / high

***FMD prevention/control intentions***

- 1. Would you vaccinate your cattle 2 times/year if the government provides the vaccine at market price (i.e. 40 birr/animal /year) ?

Yes / No

- 1. Would you vaccinate your cattle 2 times /year if the government provides the vaccine for free?

Yes / No

- 1. If it is necessary to keep your cattle isolated from other herds all the time (continuously) and to control their movements to protect your cattle from an FMD, would you do it?

Yes / No

- 1. If it is necessary to keep your cattle isolated from other herds during an outbreak of FMD and to control their movements (up to three months ) to protect your cattle from FMD, would you do it?

Yes / No

***Note: The Amharic version of this questionnaire is available up on request from the corresponding author.***
